# The Potential of Combining Tubulin-Targeting Anticancer Therapeutics and Immune Therapy

**DOI:** 10.3390/ijms20030586

**Published:** 2019-01-30

**Authors:** Alexis Fong, Amanda Durkin, Hoyun Lee

**Affiliations:** 1 Health Sciences North Research Institute, 56 Walford Road, Sudbury, ON P3E 2H3, Canada; afong@hsnri.ca (A.F.); adurkin@hsnri.ca (A.D.); 2 Biomolecular Sciences, Laurentian University, 935 Ramsey Lake Road, Sudbury, ON P3E 2C6, Canada; 3 Departments of Medicine, the Faculty of Medicine, the University of Ottawa, Ottawa, ON K1H 5M8, Canada

**Keywords:** microtubule inhibitors, immune therapy, chemotherapeutics, cell cycle dysregulation, immune cells, cytokines

## Abstract

Cancer immune therapy has recently shown tremendous promise to combat many different cancers. The microtubule is a well-defined and very effective cancer therapeutic target. Interestingly, several lines of evidence now suggest that microtubules are intimately connected to the body’s immune responses. This raises the possibility that the combination of microtubule inhibitors and immune therapy can be a highly effective option for cancer treatments. However, our understanding on this potentially important aspect is still very limited, due in part to the multifaceted nature of microtubule functions. Microtubules are not only involved in maintaining cell morphology, but also a variety of cellular processes, including the movement of secretory vesicles and organelles, intracellular macromolecular assembly, signaling pathways, and cell division. Microtubule inhibitors may be subdivided into two classes: Anti-depolymerization agents such as the taxane family, and anti-polymerization agents such as colchicine and vinka alkaloids. These two different classes may have different effects on immune cell subtypes. Anti-depolymerization agents can not only induce NK cells, but also appear to inhibit T regulatory (Treg) cells. However, different inhibitors may have different functions even among the same class. For example, the doxetaxel anti-depolymerization agent up-regulates cytotoxic T cells, while paclitaxel down-regulates them. Certain anti-polymerization agents such as colchicine appear to down-regulate most immune cell types, while inducing dendritic cell maturation and increasing M1 macrophage population. In contrast, the vinblastine anti-polymerization agent activates many of these cell types, albeit down-regulating Treg cells. In this review, we focus on the various effects of tubulin inhibitors on the activities of the body’s immune system, in the hope of paving the way to develop an effective cancer therapy by combining tubulin-targeting anticancer agents and immune therapy.

## 1. A Brief Overview of Microtubules in the Context of Mammalian Cell Cycle

The cell division cycle is a series of events leading to the formation of two mitotic daughter cells with a complete set of the entire genome for each cell [1]. The cell cycle comprises two compartments: Interphase and mitotic (M) phases. The interphase, occupying approximately 22–23 h of the typical 24 h cell cycle, can be further subdivided into G1 (gap1), S (synthesis), and G2 (gap 2) phases [1]. Large part of the cellular contents are duplicated during G1; the complete and accurate duplication of the entire genome occur during S phase; and the duplicated genome undergoes a “verification” process in G2 [1]. When cells are not in an active proliferation state, they may be arrested at a resting stage called G0 phase for a prolonged period. M phase typically lasts only 1–2 h. Like interphase, M phase can also be subdivided into various stages. The first stage, termed prophase in mammalian cells, involves chromosome condensation, and nuclear membrane breakdown, followed by duplication and moving of the centrosome toward two poles, and mitotic spindle formation [2]. The microtubule plays an essential role in this stage, particularly for centrosome movement and spindle formation. The attachment of the microtubules at opposite spindle poles allows for the chromosomes to be pulled towards the ends at the subsequent stages. During the second stage, termed metaphase in mammalian cells, chromosomes are further condensed and aligned at the cell’s equator [2]. One of the most important cell division controls called spindle checkpoint operates during this cell cycle compartment. The third stage, anaphase, discerns the separation of the sister chromatids, which is the result of the degradation of securin by separase and the shortening of microtubules [2]. The chromosomes migrate towards the spindle poles following their separation [2]. The final stage of mitosis, telophase, occurs when the chromosomes reach their respective poles, followed by cytokinesis. The optimal microtubule dynamics are critical for the smooth cell division process [3].

Microtubules are composed of heterodimeric chains of α-tubulin and β-tubulin molecules. There is a third subtype termed γ-tubulin in eukaryotes, which is needed for the nucleation of the α/β-tubulin polymerization [4]. The various interactions between the tubulin dimer in the microtubules are responsible for maintaining its tubular form as well as trafficking proteins and organelles [4]. While the microtubule is a key regulator of cell division, its dysregulation may contribute to the development of tumorous cells, as evidenced by the fact that the majority of tumor cells display aneuploidy [5]. Furthermore, tubulin mutations can also contribute to the development of chemo-resistance and tumor propagation through altered responses to cell microenvironment [5]. Some of these mutations have been identified through notable differences between tubulin isomers, tubulin post-translational modifications, and differences in the tubulin associated molecular patterns [5]. While a specific oncogenic tubulin isotype is yet to be discovered, many studies have shown that oncogenic pathways such as the AKT and ERK pathways function through the microtubule [6].

## 2. The Immune System as the Body’s Security Guard

During oncogenic activation and the subsequent cellular proliferation stage, cancerous cells may be recognized and eliminated through the body’s immune system [7]. As more mutations accumulate, pathogenic cells including cancer cells may present more cell surface antigens which in turn activate different components of the immune system, ultimately leading to their demise by the immune system [8]. Unfortunately, some cancer cells may develop special mechanisms to exploit and overcome their elimination by the immune system [8]. The good news is that the immune system can activate different subsets of mediators, which are dependent upon the presentation of a primary tumor (initial tumor) or a secondary tumor (a new tumor derived from the primary tumor) [7]. Some of these key mediators include M1 macrophages, natural killer cells (NK), neutrophils, T cells, B cells, and dendritic cells, among others [9]. This aspect will be discussed in more detail below.

## 3. Tubulin Inhibitors Effectively Target Cycling Cancer Cells

**Figure 1:**
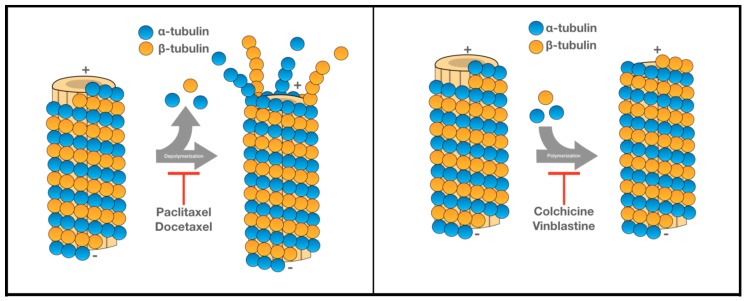
Demonstrates how the tubulin inhibitors affect the microtubules by preventing depolymerization or polymerization. Panel left illustrates the effects of paclitaxel and docetaxel (depolymerization inhibitors), while panel right illustrates the effects of colchicine and vinblastine (polymerization inhibitors).

While the immune system alone may be able to eliminate highly proliferative metastatic tumor cells, this process can be more effectively achieved when combined with other therapies. There are many different anticancer agents targeting various components of cell regulation, especially the mitotic stage of tumor cell proliferation. Although the microtubule is an effective target at different stages of cell regulation, many microtubule inhibitors are especially effective at the mitotic stage. Agents targeting tubulin may be classified into two major classes: Anti-depolymerization and anti-polymerization of the microtubule cytoskeleton (Figure 1). Among microtubule depolymerization inhibitors, taxanes are arguably the most effective compounds, and have been used effectively for the treatments of several different cancers [10]. The binding of a taxane compound to its binding-site inhibits the depolymerization of the microtubules, leading to mitotic arrest and eventual tumor cell death [11]. Paclitaxel, a first generation taxane, was initially isolated from the tree *brevifoliaI* and utilized to treat breast cancer [11]. For clinical administration of paclitaxel, nab-paclitaxel (nanoparticle albumin-bound paclitaxel) allows for a higher solubility of the drug, enhancing its delivery to patients [12]. Nab-paclitaxel also decreases the toxicity associated with paclitaxel delivery to patients [12]. Due to its high demand and scarcity of the natural sources, its semi-synthetic version docetaxel was developed [11]. Studies with tumor cell lines showed that docetaxel is a 1.3–12 fold more effective than paclitaxel [13,14]. Docetaxel, unlike paclitaxel, displays linear pharmacokinetics and is thus retained intracellularly for a longer period of time [15]. Compounds binding to the taxane-binding site may also inhibit the Bcl-2 gene activation (through phosphorylation), thus promoting apoptosis, in addition to stabilizing microtubules (Table 1) [16].

The second class of microtubule inhibitors works by inhibiting microtubule polymerization, which may be further divided into two subclasses based on their targets: The vinca-binding domain or the colchicine-binding domain. Vinca alkaloids, the prototype of the former subgroup, are originally from the periwinkle plant, *Catharanthus roseus*, and are often used to treat a variety of different neoplasms [17]. Contrary to taxanes, vinca alkaloids bind directly to the tubulin dimer, thus disrupting microtubule functions (Table 1) (Figure 1) [17]. As a result of the disruption, the mitotic spindle becomes defective, leading to a prolonged metaphase arrest [17]. Another difference is that vinca alkaloids bind rapidly to the tubulin in a reversible manner, while taxanes and colchicine site-binding compounds do not [18].

Colchicine site-binding compounds are also important microtubule polymerization inhibitor. Colchicine alkaloids, originally derived from plant *Autumn crocus*, have been well-documented for their use for the treatments of gout, inflammation, and possibly cancer [19]. Similarly to vinca alkaloids, colchicine compounds bind to the colchicine-binding site on the β-tubulin, inhibiting microtubule polymerization and leading to a prolonged metaphase arrest (Table 1) [19]. Unlike vinca alkaloids, however, colchicine binds to the tubulin in a poorly reversible manner, leading to the prevention of microtubule polymer elongation [19]. Microtubule growth arrest or microtubule depolymerization are dose dependent with a higher dose causing the latter response [19].

Having briefly described the immune system and different roles of microtubule inhibitors, the current review aims to provide insights into microtubule inhibitors in the context of the body’s immune responses. We here describe how different “classes” of tubulin-targeting agents up-regulate or down-regulate the immunomodulatory activity of T cells, NK cells, monocytes, and dendritic cells. There is an excellent possibility that the chemo-immuno combinational therapy can substantially improve the outcome of cancer treatments. To achieve this goal, it will be imperative to understand if and how anticancer chemotherapeutic agents affect the regulation of the body’s immune responses.

## 4. Regulation of T Cells

T cells are the integral part of the immune system as they are responsible for cell-mediated immune reactions [27]. Naïve T cells are immunologically not active and can only differentiate into their effector states (CD4+ or CD8+) upon the recognition of foreign antigens presented by antigen presenting cells such as dendritic cells, macrophages, and B cells [27]. T cells can take on an activated phenotype of either CD4+ helper function or CD8+ cytotoxic capabilities [28].

CD8+ cytotoxic T cells are essential for providing protection against pathogens, as they can directly kill and remove pathogen-infected cells [27]. To kill an infected cell, a cytotoxic T cell may release perforin to create pores in the plasma membrane of the targeted cell, allowing proteases to enter the cell and promote its death [27]. Cytotoxic T cells can also induce apoptotic cell-killing by activating the caspase cascade in the target cell [27]. CD4+ T cells are involved in the adaptive immune response by becoming T helper cells upon stimulation by an APC (antigen presenting cell). The activated T helper cells then can stimulate B cells or cytotoxic T cells, while secreting cytokines and chemokines to activate neighboring cells. T helper cells are further divided into Th1 and Th2 cells according to the cytokines they produce [28]. Th1 cells secrete TNF-α and IFN-γ to activate macrophages and cytotoxic T cells, which promotes the killing of intracellular pathogens [27]. Th2 cells secrete several different cytokines including IL-4, IL-5, IL-10 and IL-13 to help defend the body from extracellular pathogens [27]. The main effect of these interleukin cytokines is to stimulate B cells to create antibodies [27]. The antibodies would then bind to innate immune cells (i.e., mast cells, basophils, and eosinophils) to induce local mediators to fight off infection [27]. In addition to Th1 and Th2 subsets, there are other T helper cells that play an important role for immune responses, including Treg, Tfh, and Th17 cell types [28]. Treg cells may suppress the immune response by down-regulating the proliferation of T cells and their cytokine productions, which is important for the body to prevent the induction of autoimmune responses [29]. Treg cells are typically increased in the tumor microenvironment to inhibit the cytotoxic T cells from killing cancer cells, a typical mechanism of cancer cells evading the immune surveillance [30]. In general, T cells work to protect our bodies from infections and to eliminate foreign invaders by facilitating an effective immune response.

## 5. Responses of T Cells to Microtubule Inhibitors

The treatment of colchicine on healthy individuals results in the reduction of both T helper and cytotoxic T cell populations [31]. However, the exact mechanism of how this global decrease in the overall T cell population by colchicine is unknown, although the down-regulation of cell division cycle could certainly be one reason.

The effects of paclitaxel on T cells have been studied more in detail by several groups. For example, Vicari and colleagues demonstrated that paclitaxel substantially decreases the numbers of Treg cell numbers in the spleen of both normal and tumor-bearing mice [32]. Further investigation revealed that the level of FOXP3 expression was substantially decreased in Treg cells in response to paclitaxel, indicating a decrease in the inhibitory capacity of Treg cells [32]. As a result, Treg cell-mediated T cell proliferation was severely impaired [32]. Typically, the down-regulation of Treg cells increases the CD8+ T effector cell population, due to a decrease in the Treg-mediated suppression of CD8+ cells [32]. Surprisingly, however, paclitaxel’s ability to decrease Treg cells did not result in an up-regulation of CD8+ T cell activities [32]. Paclitaxel actually down-regulates both Treg and CD8+ T cell subtypes, probably due to the toxic effects of paclitaxel on the cycling cells [32]. This indicates that paclitaxel does not promote immune responses, but suppresses overall T cell population and thus their activities.

Mullins and colleagues investigated in a murine model whether paclitaxel-mediated immunosuppression on T cells can be reversed by IL-12 [33]. The authors found that the administration of exogenous IL-12 can not only overcome the paclitaxel-mediated suppression of CD4+ T cells, but actually promote cell proliferation [33]. This result suggests that, while paclitaxel alone reduces the T cell population, the combination of paclitaxel with IL-12 can have positive effects on T cell activities [33].

Li and colleagues (2014) found that docetaxel decreases Treg cells in patients with non-small cell lung cancer [34]. Similar to this study, Turk et al. (2004) found that docetaxel treatment resulted in the decrease of Treg cells in a mouse model, leading to an increase in cytotoxic T cells [30]. Thus, docetaxel has the potential of increasing its anti-tumor activity by promoting T cell-mediated tumor cell killing, in addition to its direct tumor cytotoxicity.

Similarly, vinblastine suppresses tumor-induced Treg cells [35]. In this case, the killing of the tumor cells appears to work by increasing the CD8+ T cell population, as vinblastine was not effective when CD8+ T cells were depleted in the host animals [35]. Therefore, vinblastine has the ability to selectively eliminate Treg cells, while not reducing the CD8+ T cell population. Thus, vinblastine can enhance the killing of cancerous cells by immune system [35].

**Figure 2:**
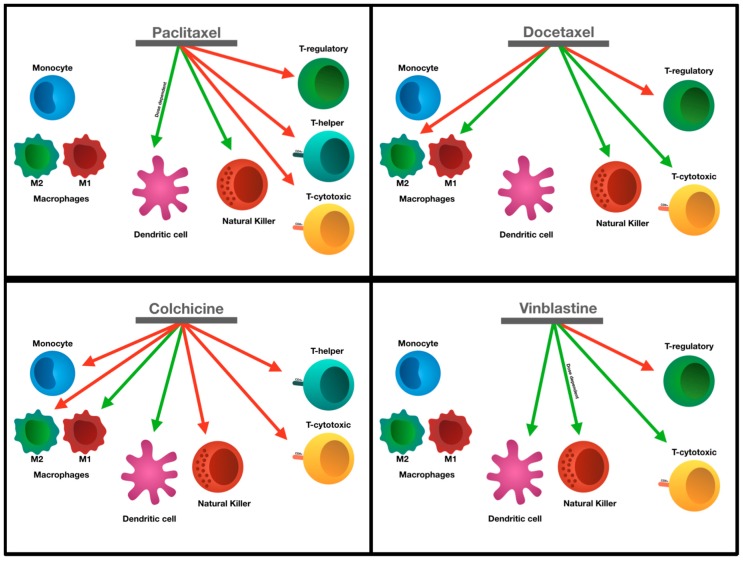
Tubulin inhibitors and their effects on the various immune cell types. Green arrows indicate the ability of the drugs to induce activation and red arrows indicate the inhibition of that immune cell type.

Taken together, certain tubulin-targeting anticancer agents have the potential of increasing their anti-tumor activities by down-regulating Treg cells (Figure 2). In most of these cases, the drugs selectively target the regulatory T cells while sparing/increasing the proliferation of cytotoxic T cells, allowing cytotoxic T cells to attack the cancer cells. Unfortunately, paclitaxel, one of the most commonly used tubulin-targeting agents, reduces both the Treg cells and the cytotoxic T cell populations. These aspects should be an important consideration for a combined cancer therapy with anti-microtubule agents and immunogenic therapeutic agents such as checkpoint blockades.

## 6. Regulation of Monocytes

Monocytes are a type of white blood cells, accounting for approximately 5% of the leukocytes in the body. They are found in circulating blood, bone marrow, and spleen [36,37]. Monocytes have many functions in the body, including maintaining homeostasis and assisting of immune defense and tissue repair [38]. As effector immune cells, monocytes circulate in the blood stream and migrate into specific sites if or when infections happen [36]. One major effector function of monocytes is to secrete inflammatory cytokines to combat foreign invaders/microbial infections [36]. Monocytes are often divided into three types based on the varying levels of CD14 and CD16 surface markers [38]. A typical monocyte has a high expression level of CD14 and absence of CD16, while a non-classical monocyte has a low level of CD14 and no expression of CD16 [38]. The intermediate monocyte is identified by a high level of CD14 with a low level of CD16 [38]. The intermediate monocyte is thought to be a subpopulation of monocytes that act during reparative processes, as they have high levels of surface markers such as CXCR4 and vascular growth factor [39].

Monocytes are also capable of differentiating into dendritic cells or macrophages, especially in response to inflammation [36]. Macrophages are phagocytic cells that can detect and engulf foreign substances, pathogens, and cellular debris via phagocytosis [40]. Macrophages detect pathogens and help activate the adaptive immune system by presenting antigens, inducing the production of inflammatory cytokines [36]. Macrophages increase inflammation and stimulate the immune system. Interestingly, however, macrophages can also be anti-inflammatory, as described below. Macrophages that induce inflammation are referred to as M1 macrophages, while the M2 macrophages decrease inflammation and promote tissue repair [40].

Monocytes and macrophages play an important role in the tumor microenvironment. Upon recruitment to tumors, monocytes may differentiate into tumor associated macrophages, which promote the initiation, progression, and metastasis of tumors [41]. Understanding the mechanism of how monocytes and macrophages behave in response to microtubule inhibitors is critically important, since relevant drugs may enhance or suppress the immune cell-mediated tumor cell killing.

## 7. Responses of Monocytes and Macrophages to Microtubule Inhibitors

Manie and colleagues (1993) found that microtubule inhibitors promote the secretion of IL-1 by human monocytes [42]. The authors also found that IL-1 expression by colchicine is due to the disruption of the microtubules, leading to the activation of protein kinase A [42]. The protein kinase A pathway is necessary but not sufficient to induce IL-1 production by disrupting the microtubules [42]. Unlike IL-1, the expression of IL-6 and TNF-α cytokines is not stimulated by colchicine [42].

A study carried out with mouse monocytes and macrophages revealed that colchicine could prevent monocyte proliferation and differentiation [43]. The authors found that the level of pro-inflammatory M1 macrophages is up-regulated, while that of anti-inflammatory M2 macrophages is down-regulated in the presence of colchicine [43]. These data demonstrate that colchicine can simultaneously induce pro-inflammatory macrophages and prevents monocyte differentiation [43]. Thus, it is clear that the contradictory effects of colchicine on inflammation and immune system should be investigated further if we want to utilize a colchicine-immune combinational therapy. Similar to colchicine, docetaxel also appears to promote the differentiation of human monocytes into pro-inflammatory M1 type macrophages, while slightly decreasing the anti-inflammatory M2 macrophages [44]. This is a contrast to paclitaxel, which does not alter the differentiation of M1 or M2 macrophages [44]. However, both of them do promote the activation of monocytes and macrophages in vitro [44]. Interestingly, the activation of monocytes by docetaxel may be relevant to its induction of IL-8 and IL-1β. The authors noted that paclitaxel can affect the levels of the cytokines only at a high concentration [44].

Taken together, colchicine effectively reduces monocytes, while inducing the pro-inflammatory M1 macrophages. Docetaxel promotes the differentiation of monocytes into M1 macrophages, whiles inducing cytokine secretion in monocytes. Paclitaxel appears to have no effect on the differentiation of monocytes and their cytokine secretion unless administered at a very high dose (Figure 2). Thus, different tubulin-targeting agents show different effects on immune responses.

## 8. Regulation of NK Cells

Natural killer cells, a specific type of lymphocyte, constitute an important component of anti-tumoral immunity with their ability to secrete various cytokines and chemokines as well as displaying cytotoxic activity [45]. Mature NK cells, which comprise 10–15% of blood lymphocytes, circulate through the blood stream to find and eliminate harmful foreign bodies [46]. Natural killer cells possess a specific phenotypic trait as they are all present with CD56 antigens on the cell surface [46]. Physiologically, NK cells produce IFN-γ, thus mediating adaptive immune responses as well as regulating antibody-dependent cytotoxicity through CD16 [46]. Natural killer cells also express receptors for MHC I molecules [45]. Importantly, NK cells have the ability to regulate a variety of cell types including dendritic cells, through the secretion of various cytokines [45,47].

Unlike T cells, NK cells do not require any previous stimulation or an adaptive immune response to elicit its killing properties [47]. NK cells possess only limited clonal expansion, which may be useful in limiting the quantity of cytokines being released in circulation [47]. In the tumor microenvironment, however, NK cell (like T cells) are subjected to the down-regulation of their receptor as a mean to circumvent their cytokine release during neoplasm development [47]. Historically, NK cells have been examined to prevent metastases without much success, even at a very high activity. This failure led to the hypothesis that NK cells do not play any major role in defense against tumors that do not circulate through the blood stream [48]. Activated NK cells, found in well-vascularized tumor microenvironments, are dependent upon the stimulation of IL-2 or IL-15 [48]. This stimulation allows NK cells to survive, proliferate, and maintain functional activity. Without continuous stimulation, NK cells may begin to undergo apoptosis by 24 hours [48]. Provided there is sufficient IL-mediated stimulation, NK cells can target cancerous cells through the activation of the NKp46 receptor. This allows the induction of IFN-γ, which leads to the expression of fibronectin [49]. Glasner et al. showed that Ncr1-l (mouse equivalent to NKp46) knock-out mice have increased neoplastic potential, although it did not affect primary tumor growth [49].

## 9. Responses of NK Cells to Microtubule Inhibitors

Kubo et al. showed that clinically relevant concentrations (nanomolar range) of paclitaxel substantially increase the NK cell-mediated cytotoxicity against the BT-474 breast adenocarcinoma cells [50]. The same study also showed that paclitaxel induces the activation of NF-κB while increasing the production of perforin, a key molecule of NK cell-mediated cytotoxicity [50]. Di Modica and colleagues examined the expression of various ligands in the BT-474 and MDA-MB361 breast carcinoma cells treated with 100 nM of docetaxel [51]. In this study, the authors found substantially increased expression of NKG2D, an important regulator of NK cell activation [51]. The authors also found that there is no phenotypic difference between NK cells in a pre-clinical mouse model before and after docetaxel treatment [51]. In contrast, 10 μg/mL of paclitaxel inhibited cytotoxicity against both NK cell-sensitive (K562 ovarian cells ) and NK cell-resistant (OV-2774) cell lines [52].

While certain taxanes enhanced NK cell activity, the same cannot be said about colchicine (see Figure 2). In a study conducted in 2013, Orange and colleagues did not find that colchicine has any substantial effects on the activity of human NK cells, although they show unusual morphology (e.g., bubbly appearance) in response to colchicine [53]. Katz et al. showed somewhat different results as they demonstrated that colchicine actually inhibits the cytotoxic effects of human NK cells [54]. A few other studies showed that colchicine does not have an effect on adhesion or synapsis formation of human NK cells [55]. Davis and his group determined that NK cell synapse formation is not compromised in the presence of colchicine in a human cell model [56]. The degranulation of human NK cells was increased four-fold in response to a low dose (0.1 μM) colchicine when compared to a high dose (10 μM of colchicine) [57]. This increase in degranulation of NK cells leads to the release of cytolytic granules, an important prerequisite for the NK cell-mediated cytotoxicity [57].

The effects of vinblastine on NK cells are somewhat controversial. Carpén and colleagues reported that, unlike colchicine and taxanes, vinblastine does not impede human NK cell function [58]. In contrast, the Markasz group found that vinblastine inhibits human NK cell-mediated tumor-cell killing, without causing changes in cellular morphology [46]. Further, these authors found that other members of the vinca alkaloid group including vincristine and vinorelbine also inhibit NK cell-mediated tumor-cell killing, albeit less effective than vinblastine [46]. Studying with a pre-clinical mouse model, the Kang group found that vinblastine can enhance or suppress the NK cell activity, depending on the doses used: 1–10 µg (per kg of body weight) enhanced NK cytotoxic activity, while 0.1 µg suppressed it [59]. Clearly, further research is required to sort out this controversy and confusion associated with the vinca alkaloid group on NK cell activities.

## 10. Regulation of Dendritic Cells

Dendritic cells are the only APC with the ability to stimulate naïve T cells, through which they can activate primary immune responses [60]. Dendritic cells are differentiated from a diverse lineage, and are found in small populations dispersed in various locations in the body, most commonly in the lymphatic regions [60]. Dendritic cells have an efficient mechanism to stimulate naïve T cells [60]. To activate T cells, dendritic cells require co-stimulators such as CD40, CD80, and CD86 [60]. While naïve T cells are the primary cell population modulated by dendritic cells, memory T cells are also effectively stimulated by them [60]. In addition, dendritic cells are important cytokine producers, ultimately helping to elicit immune responses.

Comparative phenotypic studies have revealed that dendritic cells are derived predominantly from myeloid or plasmacytoid cells [61]. Myeloid dendritic cells express CD11b, CD11c, CD13, and CD33 cell surface antigens, while plasmacytoid dendritic cells express CD123, CD303, and CD304 antigens [61].

Dendritic cells are present in the tumor microenvironment (TME), where they can interact with antigens present on dead tumor cells or nibble at living tumor cells, leading to the activation of T cells [62]. However, tumor cells have the ability of preventing antigen presentation through various mechanisms. First, tumors can alter the typical differentiation process of monocytes by differentiating them to macrophages, rather than to dendritic cells, thus preventing the activation of tumor-specific T cells [62]. Tumors can also impede dendritic cell maturation through the secretion of IL-10 [62]. A recent study demonstrated that the infiltration of CD103+CD11b dendritic cells inhibit IL-12 expression through IL-10 secretion in a breast cancer model [63]. As a result, antigen-specific T cell responses may be hindered. Tumor derived factors can also impede dendritic cell maturation [62]. Aspord et al. showed that TSLP, a cytokine and tumor derived factor, induces the expression of OX40 ligand in dendritic cells, resulting in the T-helper cells accelerating tumor development through the release of IL-4 and IL-13 [64]. These interleukins play a critical role in abrogating tumor cell apoptosis and promoting tumor cell proliferation [64]. One of the major mechanisms by which dendritic cells are impaired during anti-tumor immunity is the result of lipid accumulation in dendritic cells [63]. Dendritic cells with elevated lipid content are not able to effectively stimulate T cells or display tumor associated antigens [65].

## 11. Responses of Dendritic Cells to Microtubule Inhibitors

While paclitaxel is often used to treat breast, ovarian, and prostate cancers, its high doses have been shown to be immunosuppressive. Ferrari and colleagues demonstrated that cytotoxic levels of paclitaxel on dendritic cells can lead to a decrease in dendritic cell mobilization [65]. However, low doses of paclitaxel (200 mg per m^2^ body) seem to enhance the immunostimulatory activity of dendritic cells [66]. Paclitaxel also interacts with TLR-4 (an LPS receptor) in a murine model, thus positively effecting cell maturation and promoting an immune response [67]. John et al. demonstrated that there is a dose-dependent relationship between dendritic cell proliferation and the subsequent T-cell activation when treated with paclitaxel [68]. They found that cytokine gene expression was down-regulated and chemokine secretion was lacking in dendritic cells treated with paclitaxel, the latter may suggest a decrease in dendritic cell migration in the lymphatic system [69].

Wen et al. demonstrated the importance of functional microtubules for antigen processing by dendritic cells [69]. For example, colchicine was found to promote dendritic cell maturation and antigen-cross presentation [69]. Consistent, the treatment of B16F10 mouse melanoma cells with 2.5 μM colchicine elicited responses of CD4+ and CD8+ T cells [69]. The treatment also induced the expression of damage-associated molecular patterns and tumor-associated antigens [69]. Mizumuto, et al. demonstrated that the expression of MHC II markers was markedly increased following treatment with colchicine in the CD34+ murine progenitor cells [70]. The CD40, CD80 and CD86 co-stimulatory molecules are also up-regulated in response to colchicine [70]. Furthermore, this group also found that dendritic cells treated with colchicine show elevated levels of IL-6, IL-8 and macrophage inflammatory proteins (namely 1α and 3α), without showing the expression of TNF-α [70]. The study also demonstrated an increase in the uptake of FITC-DX, indicating an increase in endocytotic activity in dendritic cells treated with colchicine [70].

Similarly to colchicine, vinblastine induces dendritic cell maturation [71] (Figure 2). Furthermore, vinblastine also inhibits suppressor T cells in tumor microenvironment of a mouse model [71]. Tanaka et al. found that the treatment of mouse bone marrow (BM) derived dendritic cells with vinblastine (ranging from 0.1 to 1.0 μM) leads to increases in the levels of IL-12, IL-6, and IL1-β cytokines as well as CD40, CD80, and CD86 co-stimulatory molecules [72]. In addition, the same treatment of dendritic cells also leads to an increase in the level of MHC II, and consequently, an increase in T-cell stimulatory activity [72]. The authors further alluded to the mechanism by which vinblastine could potentiate dendritic cell maturation, leading them to conclude that there are direct and indirect mechanisms involved in the maturation process in response to vinblastine. The latter may occur through a secondary activation of BM-DC in vinblastine pre-treated cells [72]. Similarly to colchicine treatment, the administration of vinblastine augments the endocytotic capability of dendritic cells, but to a greater extent than colchicine [72]. Most of the pre-clinical studies investigating the effects of tubulin-targeting agents demonstrated that they can induce dendritic cells activity, as illustrated in Figure 2.

## 12. The Art of Combining Tubulin Inhibitors and Immunotherapy

Historically, much effort has been undertaken into understanding the roles and impacts of various tubulin-targeting agents in the context of cancer treatments. However, targeting tubulin alone shows only limited success. Many studies have shown the potential benefits of combination of anti-microtubule agents with other therapeutics, especially with immunotherapy [73,74]. Immunological therapeutics such as checkpoint blockades show tremendous potential for the control of tumors. However, immunotherapy alone is thus far more effective for only relatively small subpopulations of patients for certain types of cancer [73]. Under normal physiological conditions, immune checkpoints prevent autoimmunity by inhibiting dendritic cell activation of T cells [74,75]. Immune checkpoints may also act by causing T cell exhaustion at sites of inflammation [74,75]. In a way, tumor cells cleverly use this physiological phenomenon to protect them from immune surveillance mechanisms.

Essentially, all of the currently available checkpoint blockades are monoclonal antibodies that can expose tumor cells to the T cell-based immune surveillance mechanism [75]. By enhancing or eliciting immune responses that are often down-regulated in cancerous cells, tumor cells can be selectively eliminated. However, for one reason or another, this seemingly straight forward mechanism does not always work. A promising new approach may be a chemo-immuno combinational approach. There are currently >200 clinical trials involving immune checkpoint inhibitors in conjunction with chemotherapy [74].

Pembrolizumab and nivolumab are common checkpoint inhibitors used to treat non-small cell lung cancer (NSCLC). Numerous clinical trials demonstrate the combination of tubulin inhibitors with the previously stated checkpoint inhibitors for the treatment of non-small-cell lung cancer [76,77,78]. One clinical study completed by Gadgeel and colleagues, concluded that the combination of pembrolizumab with paclitaxel and carboplatin is feasible and yielded clinically significant positive results regardless of pembrolizumab dose or PD-L1 status in NSCLC patients [78]. This is one of many clinical trial studies that demonstrates the importance and clinical impact of combining tubulin inhibitors with immunotherapies for the treatment of cancer.

## 13. Concluding Remarks

As reviewed above, the combination of tubulin-targeting anticancer agents and immune therapy appears to be especially promising, as several lines of evidence suggest that agents functioning as anti-polymerization and anti-depolymerization of microtubules can enhance the body’s immune response. The next logical step would be a better understanding of the molecular mechanism about how different tubulin-targeting agents can enhance different immunotherapeutic agents in various tumor environments. Undoubtedly, this knowledge will eventually bring about highly effective cancer therapies in individual patients.

## Figures and Tables

**Table 1:** Summary of well-known tubulin inhibitors.

Microtubule Inhibitors	Binding Domains	Cancer Treatments	Mode of Action	References
Paclitaxel (nab-paclitaxel)	Taxane-binding	Breast, ovarian, prostate, lung	Anti-microtubule depolymerization leading to mitotic arrest	[12,20]
Docetaxel	Taxane-binding	Breast, non-small cell lung, androgen-independent metastatic prostate cancer	Anti-microtubule depolymerization, and attenuation of bcl-2 and bcl-xL gene expression	[21,22]
Colchicine *	Colchicine-binding	Hepatocellular & prostate cancers	Anti-microtubule polymerization. Cell cycle arrest in metaphase	[19,23,24,25]
Vinblastine	Vinca-binding	Testicular, Hodgkins and non-Hodgkins lymphoma, breast, & germ cell cancers.	Induces wedge at tubulin interface causing tubulin self-association into spiral aggregates. Anti-microtubule polymerization, & cell cycle arrest in metaphase.	[17,26]

* Colchicine is often administered for the treatment of gout as it was FDA approved for this condition in 2009. While colchicine has not yet been approved for cancer treatment, it was shown to decrease cancer incidence in male gout patients [25].
